# MRI-guided endovascular robotics: A roadmap to MRI-native systems

**DOI:** 10.1063/5.0335952

**Published:** 2026-07-09

**Authors:** Giulio Dagnino

**Affiliations:** University of Twente, Enschede, The Netherlands

## Abstract

Magnetic resonance imaging (MRI) offers potential advantages for endovascular intervention, including excellent soft-tissue contrast, multi-planar visualization, and the possibility of radiation-free guidance. Emerging robotic platforms, MRI-compatible catheter systems, and MRI-native navigation frameworks indicate that MRI-guided endovascular robotics is moving beyond isolated feasibility concepts. Yet routine MRI-guided robotic endovascular procedures remain rare. This gap is not explained by the absence of a single enabling technology, but by the interaction of tightly coupled constraints spanning instruments, imaging and feedback, navigation, and system integration. In endovascular settings, these constraints are amplified by the need for miniaturized, flexible, and steerable tools operating under limited and phase-dependent feedback. This perspective argues that progress in MRI-guided endovascular robotics requires moving beyond compatibility-driven adaptation toward MRI–robot co-design. First, the emerging landscape of representative robotic platforms, MRI-compatible and MRI-actuated catheter systems, and MRI-navigation-enabling technologies is outlined. The coupled constraints that continue to limit translation are then analyzed, showing why component-level solutions often remain fragile when integrated into full procedural workflows. Building on this framework, four research axes toward MRI-native endovascular robotic systems are proposed: MRI-native instruments, MRI-native feedback, navigation under bandwidth-limited imaging, and integrated systems built through workflow-aware validation and benchmarking. Across these axes, the discussion emphasizes the importance of miniaturized actuation, robot-aware MRI pipelines, feedback-efficient shared control, realistic simulation, benchmarking, and workflow-aware validation. MRI-guided endovascular robotics should therefore be understood as a systems integration challenge rather than a single-device problem. MRI-native robotic systems offer a concrete pathway toward safer, more precise, and radiation-free endovascular intervention.

## I. INTRODUCTION

### A. Clinical promise and the integration paradox

Cardiovascular and neurovascular diseases remain among the dominant contributors to global mortality and disability, motivating continuous innovation in minimally invasive therapies. Large-scale epidemiological analyses from the Global Burden of Disease studies quantify the persistent worldwide burden of cardiovascular disease and stroke and highlight the urgency of scalable interventional solutions.^1,2^ Endovascular intervention has become a cornerstone of contemporary treatment by enabling minimally invasive access to vascular targets, but its performance is ultimately constrained by the quality of intraoperative guidance and the dexterity of long, flexible instruments navigating tortuous anatomy.^3^ Improving guidance while preserving safe manipulation of these instruments remains one of the central challenges of modern endovascular therapy.

In current practice, most endovascular workflows depend on x-ray fluoroscopy for guidance. Fluoroscopy delivers high frame rates and intuitive visualization of radiopaque devices but provides limited soft-tissue contrast, poor direct depiction of vessel wall pathology, and exposes patients and clinical staff to cumulative ionizing radiation.^4,5^ Occupational exposure is a well-recognized concern in vascular and interventional practice, particularly for long, complex procedures, and quantitative measurements of absorbed dose to operators during endovascular procedures underscore this risk.^6^ These limitations become especially acute when navigation requires repeated device exchanges, prolonged fluoroscopy time, and extensive use of contrast agents—common in challenging neurovascular and peripheral interventions.^7^

Magnetic resonance imaging (MRI) offers an attractive alternative guidance paradigm. Unlike fluoroscopy, MRI provides excellent soft-tissue contrast, flexible multi-planar imaging, and access to functional information.^4^ From an interventional viewpoint, the key appeal is not only radiation avoidance but the possibility of visualizing anatomy and pathology that are difficult to appreciate under x-ray alone, potentially enabling safer navigation and more informed therapy delivery. The idea of intraoperative and interventional MRI is not new. Early perspectives explicitly anticipated intraoperative MRI as an enabling technology for procedures requiring continuous imaging feedback and emphasized the need for dedicated workflow and technical integration.^8^ Over time, interventional MRI matured through advances in scanner access, interactive imaging, tracking strategies, and safety engineering.

Within endovascular intervention specifically, the translational status of MRI guidance is best described as promising but not yet routine.^4^ A systematic review focusing on MRI-guided endovascular arterial interventions summarizes preclinical and clinical studies, and makes clear that MRI-guided endovascular procedures have been demonstrated but remain limited in breadth and frequency.^9^ Complementing this, a dedicated review of MRI-guided endovascular intervention methods catalogs the state of device tracking and visualization, along with barriers that impede widespread adoption.^10^ Together, these works converge on a practical conclusion: the clinical motivation for MRI-guided endovascular intervention is strong, but the current tool ecosystem and workflow integration remain insufficient for routine practice.

Robotics can be a decisive enabler in this context—particularly because endovascular navigation is a constrained task that relies on long, flexible instruments operating under limited and indirect feedback, with friction-dominated tool–vessel interactions and stringent safety margins. Reviews of robot-assisted endovascular catheterization and endovascular robotics platforms emphasize that robotics can address fundamental procedural limitations rather than simply “automate” manual motion.^4,11,12^ Specifically, robotic systems can improve precision, stability, and repeatability, supporting consistent micro-adjustments during branch engagement and device deployment; reduce operator burden through ergonomic remote manipulation; and provide a platform for assistance and shared control, where the clinician remains in command while the system stabilizes motion, manages constraints, and compensates for intermittent or phase-dependent imaging feedback.^11,12^ Concrete implementations of shared-control primitives—such as haptic feedback and dynamic active constraints—have been proposed to stabilize endovascular manipulation while preserving clinician intent.^13^ These benefits become even more relevant in MRI, where operator access is constrained by the bore and safety zones, and remote manipulation is often operationally necessary rather than merely ergonomic.

The challenge is that MRI-guided endovascular robotics is not simply a matter of placing existing robotic systems inside an MRI scanner. Instead, the MRI environment fundamentally reshapes the design space for actuation, sensing, materials, and control. The MRI environment imposes strict constraints on materials, actuation, sensing, and electronics placement, directly interacting with the demanding requirements of endovascular navigation such as miniaturization, flexibility, steerability, torque transmission, and safety. Conventional electromagnetic actuation is generally incompatible inside the bore, ferromagnetics are prohibited, and conductive structures must be carefully managed to avoid RF-induced heating and image artifacts.^14–18^ A recent systematic review of MRI-compatible robots reinforces that these restrictions persist across device classes and remain a central impediment to translation, particularly when miniaturization and dexterity are required.^17^ More recent reviews further highlight that MRI-guided robotic intervention is constrained by coupled limitations across actuation, sensing, imaging, and workflow, rather than by a single missing component.^18,19^

From the imaging side, the constraints are equally structural. MRI must balance spatial resolution, temporal resolution, and robustness to motion and device-induced artifacts. While interventional and interactive MRI approaches exist, the imaging performance required for dynamic navigation and closed-loop robotic control differs from diagnostic imaging and may vary across procedural phases (coarse navigation vs fine targeting vs therapy delivery). Reviews of real-time MRI emphasize ongoing progress but also underline that “real-time” imaging entails tradeoffs that become especially consequential when imaging is used as the primary feedback channel for control.^19,20^

Crucially, robotics and imaging constraints are interdependent. Design choices aimed at improving device localization—such as adding conductive elements for active tracking—can increase RF heating risk or artifact burden. Conversely, sequence choices that improve anatomy may reduce device conspicuity. This coupling helps explain why MRI-guided endovascular robotics has progressed incrementally and remains limited to specialized contexts despite decades of activity.

**FIG. 1.**
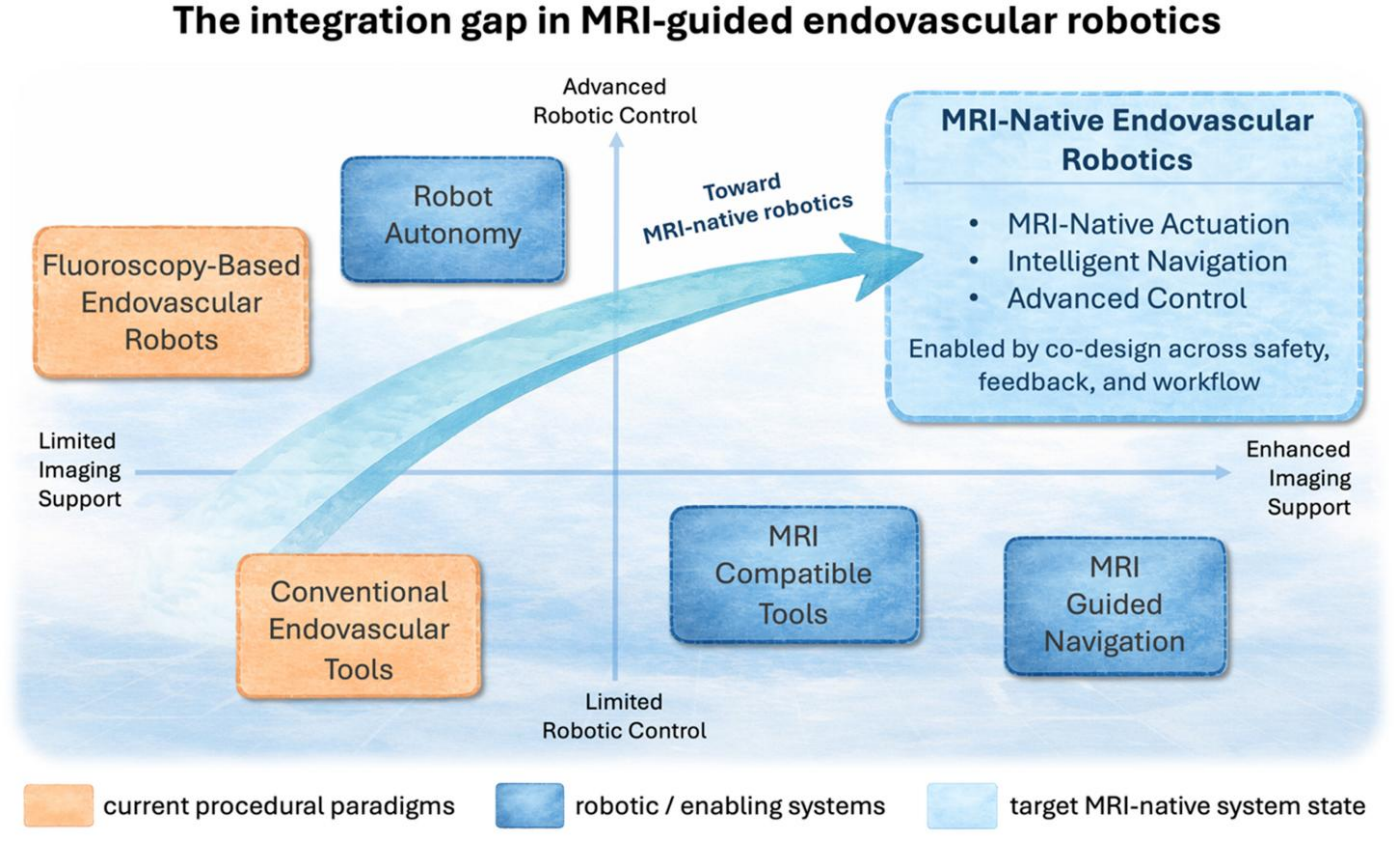
The integration gap in MRI-guided endovascular robotics. Current endovascular intervention is largely performed using pre-shaped instruments, while existing robotic platforms remain predominantly dependent on fluoroscopy-based workflows. MRI-guided procedures offer the prospect of richer anatomical feedback and improved guidance, but this potential cannot be realized by simply transferring existing tools and robotic systems into the MRI environment. Instead, MRI-guided endovascular robotics requires the co-design and integration of actuation, navigation, control, safety, and workflow within the constraints of MRI. The figure highlights the gap between existing approaches and the target state of MRI-native endovascular robotics, in which these elements are developed as an integrated system rather than as isolated components. The color coding distinguishes current procedural paradigms, robotic or enabling systems, and the target MRI-native system state.

This Perspective argues that progress in MRI-guided endovascular robotics requires moving beyond compatibility-driven design toward MRI–robot co-design. Unlike existing reviews^4,10,16,19,21^ that survey MRI-compatible robots, interventional MRI methods, flexible instruments, or endovascular robotic systems separately, this perspective focuses specifically on MRI-guided endovascular robotics as a coupled systems problem. Its contribution is not an additional inventory of devices, but a unifying framework that links the emerging system landscape to the interdependent constraints limiting translation and organizes these into an actionable roadmap centered on four coupled axes: instruments, feedback, navigation, and integration. In this sense, the Perspective moves beyond descriptive review toward translational guidance by identifying MRI–robot co-design as the principle needed to advance toward MRI-native endovascular systems. To support this shift, it (i) analyzes the coupled constraints that currently limit the field, (ii) defines what MRI–robot co-design means in practical engineering terms, and (iii) outlines research axes that can guide development toward MRI-native endovascular robotic systems. Figure 1 conceptually positions the current field and illustrates the integration gap between fluoroscopy-based robotics, MRI-guided procedures, and the transition toward MRI-native endovascular robotic systems.

### B. Clinical scenarios and requirements

To avoid a generic “MRI is better” narrative, it is useful to make the clinical pull explicit. The systematic review of MRI-guided endovascular arterial interventions^9^ and the review of MRI-guided endovascular methods and future potential^10^ highlight that the value of MRI guidance is particularly tied to situations where soft-tissue visibility, vessel-wall context, or physiological information change clinical decision-making, and where radiation avoidance is meaningful. Two exemplary clinical scenario families illustrate the requirements MRI-guided robotics must satisfy:

(i) *Neurovascular navigation and therapy delivery.* Neurovascular procedures typically involve tortuous anatomy, small diameters, and proximity to fragile structures. Under fluoroscopy, vessel-wall context and soft-tissue targets are poorly visualized, and navigation can be sensitive to subtle device–vessel interaction. MRI can offer soft tissue contrast and multi-planar views that may improve situational awareness, while avoiding radiation during potentially long procedures.^9,10^ The robotic requirements here emphasize miniaturized steerability with minimal exchange, stable micro-motion control near critical anatomy, robust localization under intermittent imaging, and strict safety margins.
(ii) *Peripheral and visceral embolization/targeted delivery.* MRI-guided embolization feasibility has been demonstrated in selective settings, and MRI could support improved assessment of target regions and post-treatment endpoints (depending on application).^9^ The requirements emphasize navigation under flow and patient motion, robust depiction of anatomy and device, and stable deployment/verification phases.

Across these scenarios, a core requirement set emerges: miniaturized steerability, predictable safety under MRI RF fields, sufficiently robust state estimation, and workflow integration. These requirements cannot be met reliably through compatibility-driven adaptation alone.

At the same time, MRI should not be assumed to be universally superior for all endovascular procedures. In many routine settings, fluoroscopy remains the most practical imaging modality because it provides high temporal resolution, established workflow integration, and broad clinical availability. Likewise, CT- or ultrasound-guided approaches may already provide sufficient anatomical or procedural information at a lower cost and with less workflow burden in selected applications. The strongest case for MRI-guided endovascular robotics therefore lies not in replacing all existing image-guided interventions, but in enabling procedures where soft-tissue context, vessel-wall information, physiological imaging, or radiation avoidance provide a meaningful clinical advantage that cannot be obtained as effectively with current alternatives.

## II. EMERGING MRI-GUIDED ENDOVASCULAR ROBOTIC SYSTEMS

MRI-guided endovascular intervention should no longer be viewed as a speculative future concept, but as an emerging technological direction supported by a growing ecosystem of robotic platforms, steerable MRI-compatible catheter systems, and MRI-native navigation and tracking frameworks. This broader trajectory is reflected not only in reviews focused specifically on MRI-guided endovascular intervention,^9,10^ but also in recent reviews of endovascular robotics, MRI-compatible flexible instruments, MRI-guided robotic intervention, and robotically steerable guidewires.^4,16,18,19,21^ Collectively, these reviews show that the field is advancing through converging developments in robotic manipulation, MRI-compatible instrumentation, imaging-guided feedback, and navigation assistance rather than through a single dominant platform.

More importantly, integrated or near-integrated systems already exist. As summarized in Table I, the current landscape includes MR-compatible and MR-safe robotic catheter manipulation platforms, MRI-guided robotic cardiovascular intervention systems, MRI-actuated or MRI-compatible steerable catheter concepts, and MRI-native feedback systems based on active tracking, passive tracking, or software-defined navigation. Representative examples include the MR-safe endovascular robotic platform family developed from the dual-modality fluoroscopy/MRI system to later MR-safe endovascular robotic validation studies,^22–24^ active catheter tracking with automated slice positioning^25^ and wireless active catheter visualization^26^ as early MRI-native feedback systems, and more recent MR-based navigation and passive tracking frameworks for robot-assisted endovascular procedures.^27,28^ Beyond these, distinct platform lines such as compact telerobotic catheter navigation systems,^29^ robotic-assisted real-time MRI-guided TAVR (Transcatheter Aortic Valve Replacement),^30^ MR-safe robotic manipulators for intracardiac catheterization,^31^ and MRI-guided robotic cardiac catheter systems with shape tracking and feedback control^32^ further indicate that integrated MRI-guided catheter-intervention systems are already emerging.

**TABLE I.** Representative MRI-guided endovascular robotic, catheter, and MRI-navigation-enabling systems. This table summarizes representative systems that collectively illustrate the emergence of MRI-guided endovascular robotics as a developing technological field rather than an isolated set of feasibility studies. The listed entries span integrated robotic platforms, MRI-compatible or MRI-actuated catheter concepts, tracking and navigation-enabling systems, and preclinical integration tools. Together, they show that multiple building blocks already exist—including MR-safe robotic manipulation, MRI-native feedback and tracking, steerable MRI-compatible devices, and software-defined navigation frameworks—but that these systems remain heterogeneous in procedural target, level of integration, and validation stage. This landscape highlights both the growing momentum in the field and the persistent integration gap motivating MRI–robot co-design.

System/platform	Type	Target application	MRI role	Validation stage	Main remaining limitation
Active catheter tracking with automated slice positioning^25^	Tracking/guidance system	Intravascular catheter procedures	Active tracking, scanner interaction, automated slice positioning	Preclinical	Enables MRI-native feedback, but not robotic manipulation
Wireless active catheter visualization^26^	Tracking/guidance system	Catheter visualization during MRI-guided procedures	Wireless active visualization	Preclinical	Visualization support only; no manipulation capability
Active steering-coil intravascular catheter^40^	MRI-actuated catheter system	Intravascular navigation	Active steering in MRI	Bench/preclinical	Heating and wiring constraints
Motion-adapted catheter navigation/visualization framework^41^	Navigation/visualization system	MRI-guided catheter navigation	Real-time MRI visualization and navigation support	Preclinical	Not an integrated robotic platform
Compact telerobotic catheter navigation systems^29^	Robotic catheter platform	MRI-guided catheter navigation	MR-compatible remote manipulation	Bench/phantom/preclinical	Early platform with limited integrated feedback
Robotic-assisted real-time MRI-guided TAVR^30^	Integrated robotic intervention system	Transcatheter aortic valve replacement	Real-time MRI-guided robotic assistance	Phantom + *in vivo* swine	Procedure-specific platform
MRI-safe robotic manipulator for MRI-guided intracardiac catheterization^31^	Robotic catheter platform	Intracardiac catheterization	Robot-assisted catheterization in MRI	Bench/phantom/preclinical	More intracardiac than broadly endovascular
MRI-safe endovascular robotic platform CathBot^22,23^	Evolving endovascular robotic platform family	Endovascular intervention	Dual-modality then MR-safe robotic endovascular manipulation	Phantom + *in vivo* swine	Strong direct endovascular MRI–robotics lineage, but still preclinical
MRI-guided robotic cardiac catheter with shape tracking and feedback control^32^	Robotic catheter platform with closed-loop feedback	Cardiac catheterization/PVI (Pulmonary Vein Isolation)simulator	MRI-guided shape tracking and feedback control	Simulator in MRI	Application-specific, limited broader procedural validation
Steerable and MRI-compatible cardiac catheter^33^	MRI-compatible steerable catheter	Cardiac catheterization/AF (Atrial Fibrillation) treatment	MRI-compatible steerable catheter	Bench/device development	Limited workflow-integrated MRI validation
Heat-mitigated MRI-driven microcatheter^34,35^	Emerging MRI-driven catheter concept	Microcatheter steering	MRI-driven actuation with heating mitigation	Bench/preclinical	Promising MRI-driven actuation concept, but limited catheter-workflow integration
MRI-gradient pulling of a tethered robot^38^	Emerging MRI-relevant robotic concept	Soft magnetic continuum/tentacle robot	MRI-relevant magnetic actuation and shape-forming design principles	Robotic concept	Not an MRI-guided endovascular system; limited direct validation for catheter-/guidewire-based intervention
Multi-selective catheter for MRI-guided endovascular interventions^36^	MRI-compatible catheter device	Endovascular catheterization	MRI-compatible selective catheter	Bench/preclinical	Not robotic; limited integrated control
MRI-gradient/ferromagnetic-tip steering systems^37^	MRI-actuated catheter/guidewire concept	Catheter/guidewire steering	MRI magnetic field/gradients used for steering	Bench/preclinical	Limited integrated workflow validation
MRI-based navigation for robot-assisted endovascular procedures^27,28^	Navigation/workflow framework	Robot-assisted endovascular intervention	Real-time MRI, segmentation, passive tracking, visual/haptic guidance	Framework/preclinical	Promising MRI-guidance for robotic systems, but static models and low framerate
Simulation environment for robot-assisted endovascular interventions^39^	Preclinical integration/benchmarking tool	Robot-assisted endovascular intervention	Simulation for development and benchmarking	Simulation	Not itself an MRI-guided intervention system

This broader system landscape also includes MRI-compatible or MRI-actuated steerable catheter concepts that are not yet fully integrated robotic platforms but remain highly relevant to the transition toward MRI-native endovascular robotics. Examples include steerable and MRI-compatible cardiac catheters for atrial fibrillation treatment,^33^ MRI-driven microcatheter concepts with heating mitigation,^34,35^ MR-guided selective catheter systems for endovascular intervention,^36^ and MRI-gradient and ferromagnetic-tip steering systems.^37^ In parallel, emerging MRI-relevant robotic concepts such as magnetically actuated shape-forming soft continuum robots suggest additional design directions for future catheter-based MRI-native systems, although these remain further from direct endovascular translation.^38^

At the same time, the landscape remains heterogeneous. Existing systems differ substantially in procedural target, imaging role, actuation strategy, level of robotic integration, and validation stage. Some systems focus primarily on robotic manipulation; others address steerability or MRI-safe actuation at the device level; still others provide MRI-native tracking, visualization, or navigation layers that can work alongside robotics. Simulation environments for robot-assisted endovascular intervention^39^ add a further layer by enabling benchmarking and integration studies before transfer to phantoms or in-scanner validation. Taken together, the systems summarized in Table I provide stronger evidence that MRI-guided endovascular robotics is already emerging beyond isolated feasibility concepts, while also showing that current platforms remain fragmented, application-specific, and only partially integrated into realistic procedural workflows. This emerging but heterogeneous platform landscape motivates the coupled-constraints analysis in Sec. III C

## III. COUPLED CONSTRAINTS

MRI-guided endovascular robotics is constrained by coupled limitations that span actuation and materials under electromagnetic constraints, RF safety, sensing and tracking under MRI restrictions, imaging tradeoffs under interventional time pressure, and workflow constraints created by the scanner environment. Reviews across MRI-compatible robotics and MRI-guided intervention repeatedly converge on the same conclusion: improvements made in isolation rarely translate into robust system-level performance because design choices in one domain directly impose constraints on the others.^14–18^

A useful way to interpret the field is therefore not “what robots or tools exist,” but “what system constraints repeatedly prevent clinical robustness.” These constraints are especially severe in endovascular settings because the core tools are long, flexible, miniaturized, and often include conductive reinforcement for torque transmission and pushability.^9,10^

### A. Instruments in MRI: Actuation, materials, and safety

MRI imposes restrictions via the static magnetic field (B_0_), rapidly switching gradients, and RF excitation. Conventional electromagnetic motors, ferromagnetic materials, and unmitigated conductive elements in or near the bore are problematic due to magnetic forces/torques, imaging artifacts, and safety hazards (e.g., RF heating).^14–18^ These restrictions do not simply exclude a component class; they force architectural redesign of how energy is delivered, how motion is generated, and where electronics can be placed.

For many MRI robots, a standard workaround is remote actuation, i.e., placing active components outside the imaging region and transmitting power via pneumatic/hydraulic lines, tendons, shafts, or other mechanisms. That approach can work for rigid targeting devices and biopsy systems, but endovascular robotics amplifies the drawbacks because endovascular navigation demands high dexterity at small diameter plus predictable distal behavior under significant frictional and anatomical uncertainty.

Pneumatics are attractive because they are intrinsically immune to electromagnetic interference and can be made MR-safe. A practical example is CathBot, a pneumatically actuated MR-compatible robot for endovascular intervention.^22–24^ However, the control of pneumatic systems becomes challenging with long transmission lines and nonlinearities introduced by compressibility and friction. These issues were explicitly addressed in the design/control of a 1-DOF (Degrees of Freedom) MRI-compatible pneumatically actuated robot with long transmission lines.^42^ That study is important not because it is endovascular, but because it highlights a recurring barrier: control bandwidth and precision degrade with transmission distance, a problem that becomes more severe as one tries to miniaturize and extend tools deeper into the body.

Hydraulic actuation can improve stiffness and controllability compared to pneumatic approaches,^43,44^ yet it introduces complexity (seals, leakage risk, pressure regulation). A compact hydraulic driving robot for intraoperative MRI-guided neurosurgery illustrates the direction.^43^ At the catheter/instrument concept level, multi-degree-of-freedom hydraulic pressure-driven “safety active catheters” demonstrate how fluidic power can be distributed to achieve complex distal behavior. Yet translating these concepts into clinically practical endovascular tools requires solving miniaturization, sterilization, reliability, and integration challenges at a much harsher scale than many MRI biopsy and targeting platforms.

Piezoelectric and ultrasonic actuation have been explored as routes to higher bandwidth motion under MRI constraints. A plastic-encased resonant ultrasonic piezoelectric actuator has been demonstrated and experimentally validated for MRI-guided surgical robots.^44,45^ Hybrid pneumatic–piezoelectric teleoperation systems (including haptic feedback) show additional integration directions.^46^ These works are relevant because they illustrate a recurring systems trade-off: higher actuation bandwidth comes with integration requirements (placement, shielding, and power delivery) that interact with imaging. At the instrument level, piezoelectric actuation has enabled MRI-guided closed-loop targeting with concentric tube continuum robots.^16^ This is one of the clearest examples that closed-loop MRI-driven robotic control is feasible in principle—yet it also highlights the scaling gap between continuum/needle-like architectures and sub-millimetric endovascular guidewires, where packaging, bending stiffness, and friction behave very differently.

A defining—and endovascular-specific—constraint for any instrument architecture in MRI is RF safety. RF heating is not a secondary compliance item. It is a first-order design constraint for elongated devices. RF heating of MRI-assisted catheter steering coils has been measured experimentally, demonstrating that conductive steering structures can generate clinically relevant heating.^47^ A closely related concern is resonant heating around intravascular guidewires due to coupling with RF fields.^47^ What makes this especially constraining for endovascular robotics is that key mechanical requirements (torque transmission, pushability, and kink resistance) are commonly achieved using metallic reinforcement, braided structures, or long conductive elements. Anything that improves mechanical performance can worsen RF coupling behavior, and vice versa. The requirements are physically entangled. Comparative safety evaluations of an MR-compatible guidewire prototype vs a standard nitinol guidewire further emphasize that heating mitigation must be treated as a primary design objective rather than a final validation step.^48^

While magnetic actuation has proven effective in fluoroscopy-guided endovascular robotics (e.g., external magnets^49–52^ or magnetically guided tools^53–56^) it is generally unsuitable inside clinical MRI due to the strong static field, safety constraints, and interference with imaging. MRI-compatible magnetic actuation approaches have been explored using embedded microcoils^40,57^ or Lorentz-force concepts that leverage the scanner's axial field to generate distal torque and bending.^58^ However, these strategies typically require substantial currents and wiring, introducing heating risks (similar to SMA (Shape Memory Alloy)-based approaches) and potential RF-induced heating along the shaft.^34^ Demonstrations using strong-field systems (e.g., 7 T) to steer magnetized guidewires illustrate feasibility but remain limited by magnet size and the non-routine clinical use of ultrahigh-field scanners.^35,38^ Overall, magnetic approaches highlight the trade-off between controllability, MRI safety, and miniaturization, reinforcing the need to co-design actuation strategies with these constraints.

### B. Tracking and imaging tradeoffs

A core challenge for MRI-guided robotics is state estimation: the robot needs tool pose (and ideally shape) at adequate rate and reliability. Active tracking techniques can provide high-quality localization, but they introduce conductive pathways and add system complexity. Phase-field dithering for active catheter tracking is one example of a tracking method that addresses the real-time localization problem at the imaging level.^59^ MR-guided intravascular procedures have used active tracking coils for real-time parameter control and automated slice positioning.^25^ Wireless active catheter visualization has also been demonstrated as a way to reduce tethering constraints.^26^

Active tracking has two recurring drawbacks in endovascular robotics: (1) conductive elements can increase RF heating risk, and (2) cabling and packaging can compromise mechanical performance and miniaturization. So even when active tracking is technically possible, it may be mechanically and safety-limited for small-diameter endovascular tools.

Passive markers and image-based tracking remain important alternatives. Sequence- and marker-dependent visualization is repeatedly emphasized in MRI endovascular review literature.^9,10^ Passive markers can avoid conductive cabling but depend on MRI sequence settings and may introduce susceptibility artifacts or obscure anatomy. Image-processing-based 3D real-time tracking of MRI-compatible devices illustrates another approach, but it inherits acquisition/reconstruction constraints and can be sensitive to motion and artifact variability.^60^ Because MRI feedback can be intermittent and phase-dependent, non-electromagnetic sensors can complement MRI. Fiber-optic force sensors for MRI-guided interventions have been reviewed comprehensively, highlighting MR-safe sensing that does not perturb imaging and can support state estimation when imaging is optimized for anatomy rather than device localization.^61^

AI-based methods may also help mitigate some of these tradeoffs, particularly when MRI is used not only for visualization but also as a feedback source for navigation and control. For example, learning-based reconstruction, segmentation, and tracking approaches could be used to enhance device conspicuity, estimate tool state from sparse or lower-quality image updates, or prioritize image content that is most relevant for control rather than for uniform anatomical display. In this context, AI (Artificial Intelligence) could support faster or more task-specific feedback by identifying control-relevant regions, predicting likely device location between updates, or adapting image-processing pipelines to different procedural phases.^62,63^ At the same time, such approaches introduce their own translational challenges, including dependence on large and representative training datasets, sensitivity to domain shift, limited interpretability, and the need for safety-aware validation before deployment in image-guided intervention. For MRI-guided endovascular robotics, AI is therefore best viewed not as a replacement for imaging or sensing, but as a complementary layer that may help make intermittent and phase-dependent MRI feedback more usable for navigation and control.

These tracking and sensing constraints are inseparable from imaging tradeoffs. Interventional MRI requires low latency, robustness to motion, and tolerance to device presence. Real-time MRI has advanced significantly, but still faces intrinsic tradeoffs between spatial resolution, temporal resolution, SNR (Signal-to-Noise Ratio), and artifact robustness.^20^ Parallel imaging has enabled major acceleration advances, but introduces reconstruction and noise-amplification tradeoffs that matter when images are used as a control signal.^64^ In endovascular contexts, this trade-space becomes even more complex because procedural phases have different requirements: navigation prioritizes update rate and visibility; branch engagement prioritizes local precision and reduced artifact ambiguity; therapy deployment prioritizes stability and verification. MRI sequences and reconstruction pipelines may need to shift dynamically, but dynamic shifting itself complicates control and workflow.

### C. Workflow and the integration gap

One reason MRI-guided robotic endovascular procedures remain uncommon is that the scanner environment imposes constraints on access, staffing, ergonomics, and procedural choreography. These factors are often treated as “implementation details,” but they shape feasibility as strongly as actuation physics.

MRI bore access limits the operator's ability to manipulate instruments directly and constrains placement of robotic hardware near the patient. Many MRI-compatible robotic systems therefore emphasize remote operation, separating actuation and control components from the bore.^14–18^ However, remote operation must be reconciled with endovascular demands such as fine tactile judgment and rapid response during complications.

A further, often underappreciated, barrier is the limited accessibility of MRI systems themselves for robotics research and development. In practice, scanner time is expensive and typically shared between clinical and research activities, which restricts opportunities for iterative engineering development and system-level testing. Equally important, software access is often constrained by vendor-specific interfaces and closed control environments, making it difficult to implement, compare, and reproduce integrated imaging–robot workflows across sites. This challenge is compounded by heterogeneity across MRI manufacturers, sequences, and software ecosystems, which complicates the development of transferable solutions and benchmarking protocols. From a translational perspective, wider availability of research-oriented MRI interfaces, more open software integration pathways, and reproducible cross-platform testing environments would substantially accelerate development, adoption, and comparison of MRI-guided robotic systems.

Workflow integration also intersects with imaging: scanning protocols must support device localization and anatomy visualization under realistic time constraints, while maintaining safety margins for RF heating and artifacts.^9,10^ Because endovascular interventions include phases that differ in imaging needs, “one sequence fits all” approaches can create either excessive latency (if tuned for high resolution) or inadequate targeting precision (if tuned for speed).^20,64^ In short, the scanner is not just an imaging sensor; it is a constrained physical environment in which the entire clinical–robotic system must operate.

In this context, robot-aware feedback refers to feedback that is designed around the informational and operational needs of robotic navigation and workflow, rather than around anatomical imaging alone. In practice, this means that MRI acquisition, reconstruction, tracking, and complementary sensing are evaluated according to whether they provide control-relevant and workflow-relevant outputs—such as device localization, local visibility, confidence in-state estimation, and predictable latency—at a level that can support safe manipulation and coordinated procedural execution. The key distinction is therefore not simply between “better” and “worse” imaging, but between imaging and sensing strategies that are informative for robotic action and those that are informative only for observation. In this sense, robot-aware feedback includes not only MRI-based visualization, but also hybrid combinations of MRI, tracking, and MR-compatible sensing that together provide usable feedback for navigation, control, and system integration.

Practical MRI–robot integration challenges become visible only at the system level, where actuation, workflow choreography, and tool–imaging interactions must be managed simultaneously. MRI-safe endovascular robotic platforms evaluated in realistic experimental settings illustrate both feasibility and the operational constraints imposed by MRI access, device visibility, and safety margins.^22,23^

A compatibility-driven approach (“make a robot safe in MRI” + “make sequences tolerate the device”) has enabled important demonstrations and continues to generate valuable components.^14–18^ However, systemic coupling creates fragility: adding coils for tracking can potentially increase RF heating risk and alter artifact fields; increasing image update rate will lose spatial detail needed for precise maneuvers; improving torque transmission mechanically can worsen MRI visibility and safety interactions.^9,10,47,48^

**FIG. 2.**
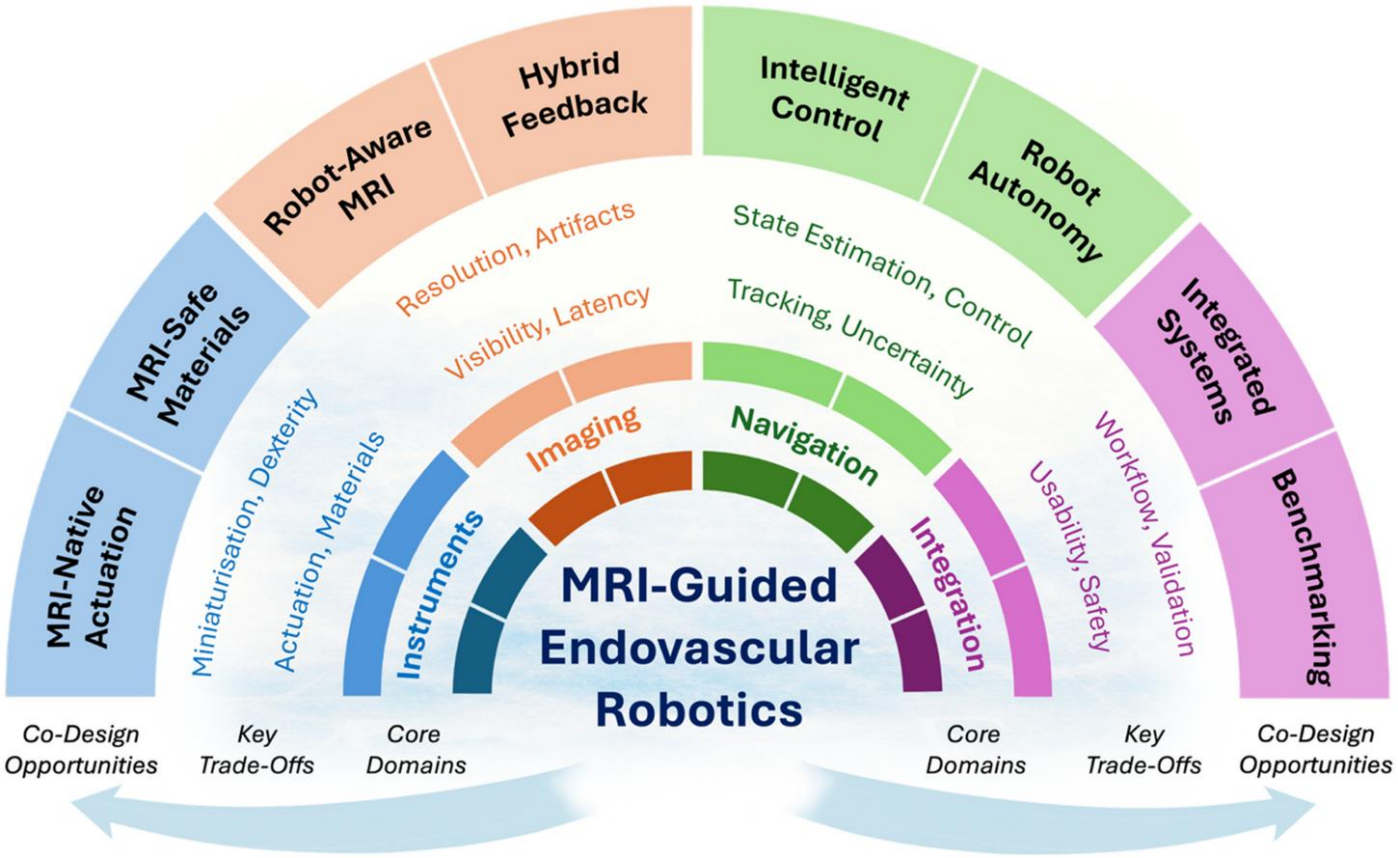
Coupled domains, key dimensions, and co-design opportunities in MRI-guided endovascular robotics. MRI-guided endovascular robotics is shaped by four coupled domains: instruments, imaging, navigation, and integration. For each domain, the middle ring highlights key technical dimensions that currently constrain translation, while the outer ring identifies corresponding opportunities for MRI–robot co-design. This framing emphasizes that progress will depend on coordinated advances in MRI-native actuation and materials, robot-aware feedback, intelligent navigation, and benchmarking toward integrated systems.

A transition toward MRI**–**robot co-design is not “nice to have”; it is a necessary response. As summarized in Fig. 2, these limitations are best understood as a coupled constraint system organized around four interdependent domains—instruments, imaging, navigation, and integration—each associated with key technical dimensions and corresponding co-design opportunities.

## IV. FROM COMPATIBILITY TO CO-DESIGN

Secs. (I, II, and III) argue that MRI-guided endovascular robotics is limited not by a single missing component, but by a coupled constraint system. This coupling explains why the field has achieved important feasibility demonstrations yet has not transitioned to routine MRI-guided robotic endovascular workflows at scale.^14–18^ The next step requires an architectural and methodological change.

### A. Limits of compatibility

Compatibility-driven development typically follows a sequential pattern: select an MR-safe actuation method (often pneumatic/hydraulic),^42–44,65^ place sensitive components outside the imaging zone,^14–18^ add tracking (active coils, passive markers, or image processing),^25,26,59–61^ and validate RF safety and artifacts after integration.^47,48^ This paradigm has been essential. Early work on MRI-compatible needle insertion manipulators for stereotactic neurosurgery^66^ and later workflow-driven demonstrations, such as robotically assisted long-bone biopsy under MRI imaging,^67^ illustrate meaningful translational progress.

However, endovascular robotics is different in two ways. First, the instrument is long and flexible: its geometry and materials simultaneously determine mechanical performance, RF coupling behavior, and artifact profiles.^9,10,47,48^ Second, MRI feedback is not a neutral measurement channel: acquisition choices trade temporal resolution, spatial detail, and robustness, and the device itself can influence what is visible.^20,64^ As a result, “bolt-on” tracking or imaging rarely yields control-grade feedback across all procedural phases.

### B. What co-design means

MRI–robot co-design is a methodology in which actuation, sensing, control, and MRI acquisition/reconstruction are co-optimized under shared performance objectives, rather than sequentially integrated under compatibility constraints. In practice, this requires four shifts:

1. **RF and artifact behavior become design variables, not *post hoc* constraints.** RF heating can occur in catheter steering structures and guidewires via RF coupling,^47^ and guidewire prototypes require dedicated RF safety engineering.^48^ Under co-design, mechanical choices (conductive reinforcement, coil placement) are made together with electromagnetic mitigation strategies and evaluated under realistic imaging conditions.
2. **Tracking and imaging are designed to support control, not only visualization.** Active tracking coils have enabled automated slice positioning and real-time parameter control,^25^ wireless active catheter visualization reduces tethering constraints,^26^ and phase-field dithering improves tracking robustness.^59^ Co-design treats tracking method selection and RF safety mitigation as a coupled optimization problem.
3. **Control architectures are designed for phase-dependent, bandwidth-limited feedback.** Even with real-time MRI advances,^20^ feedback regimes vary across procedural phases. Co-designed systems explicitly support navigation-phase control, targeting-phase precision, and deployment-phase verification, and this influences hardware, tracking, and workflow choices.
4. **Design decisions are evaluated at the system level.** Compatibility-driven work tends to validate subsystems independently. Co-design evaluates integrated performance: stable navigation and accurate targeting under representative sequence settings, with safety margins, within a plausible workflow.

This system-level evaluation perspective is supported by comparative analyses of MRI compatibility of robot actuation techniques, which emphasize that compatibility and performance must be assessed systematically.^68^ Integrated systems for MRI-guided prostate intervention similarly illustrate what it looks like to evaluate an actuated MRI-compatible robotic system in an imaging-driven workflow.^69^

### C. MRI-native systems

The literature contains partial co-design examples. Closed-loop MRI-guided targeting with piezoelectric actuation,^18^ pneumatic systems explicitly controlling long-line dynamics,^42^ active tracking with automated slice positioning,^25^ and image-processing-based real-time tracking.^60^ However, these are not yet a shared paradigm for endovascular robotics. System-level endovascular demonstrations also point toward co-design principles in practice, combining robotic manipulation with imaging constraints and procedural workflow considerations during validation.^22,23^

“MRI-native” is a meaningful term only if it conveys a concrete distinction: MRI-compatible systems operate without violating constraints; MRI-native systems are architected such that actuation, sensing, control, and imaging are inherently structured around MRI physics and endovascular workflow. Section V is framed as research axes enabling integrated, co-optimized systems rather than isolated “future technologies.”

## V. A ROADMAP TOWARD MRI-NATIVE ENDOVASCULAR ROBOTICS

**FIG. 3.**
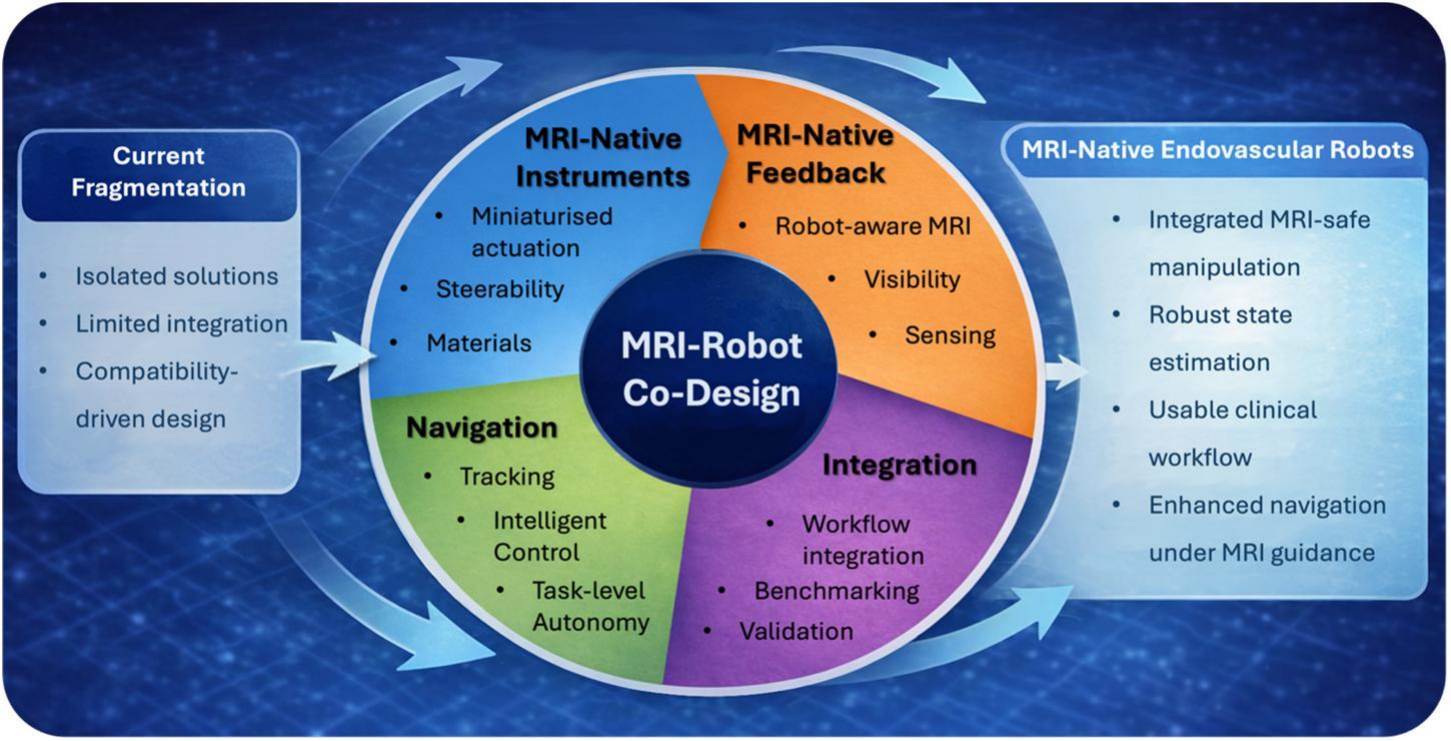
Research axes toward MRI-native endovascular robotics. The figure summarizes the transition from the current fragmented landscape of MRI-guided endovascular robotics toward integrated MRI-native systems. The left panel highlights the present state of fragmentation, characterized by isolated solutions, limited integration, and compatibility-driven design. The central wheel organizes the four main research axes identified in this perspective—MRI-native instruments, MRI-native feedback, navigation, and integration—showing the coordinated advances required across actuation, feedback, control, and validation. The right panel illustrates the targeted outcome of this co-design process: MRI-native endovascular robotic systems capable of integrated MR-safe manipulation, robust state estimation, clinically usable workflow, and enhanced navigation under MRI guidance.

The analysis developed in Secs. (I, II, III, and IV) implies that progress will not come from isolated technological advances but from coordinated development across multiple interacting system components. The following research axes define a practical roadmap toward MRI-native endovascular robotic systems. These axes are not independent technology tracks; rather, they represent tightly coupled design domains that must be addressed jointly through MRI–robot co-design. Figure 3 summarizes these research axes and their convergence toward MRI-native endovascular robotic systems.

### A. MRI-native instruments

Progress requires instrument architectures that simultaneously satisfy MRI safety, miniaturization compatible with endovascular diameters, clinically meaningful steerability and stiffness modulation, and controllability under bandwidth-limited imaging. Continuum mechanics modeling provides a foundation for thinking about remote transmission and compliance.^70^

Stiffness modulation is a particularly important enabler. A submillimeter continuous variable stiffness catheter demonstrates compliance control at clinically relevant scale,^54^ and variable-stiffness continuum devices for minimally invasive surgery show stiffness tuning while maintaining maneuverability.^53^ In MRI settings, stiffness modulation can stabilize interaction under intermittent imaging feedback and influence imaging behavior through geometry/material changes. Active stiffness tuning in a spring-based continuum robot for MRI-guided neurosurgery shows that stiffness modulation can be co-addressed with MRI integration.^71^

A defining constraint for MRI-native instruments is RF safety. RF heating is central for elongated devices: steering coils and guidewires can heat via RF coupling,^47^ and comparative guidewire safety studies show why heating mitigation must be designed in from the start.^48^ MRI-native development should normalize safety reporting and conditions and standardize testing and labeling.^72^

Finally, emerging soft actuation approaches may offer alternative tradeoffs between miniaturization, compliance, and integration complexity, but must be co-designed with RF safety, tracking, and imaging regimes. MRI compatibility of silicone-made contractile dielectric elastomer actuators provides a foundational demonstration of feasibility.^73^ Work toward broad optimal output bandwidth in dielectric elastomer actuators suggests performance progress.^74^ The roadmap point is not to select one “winner,” but to treat soft transduction as candidates within a co-design space.

### B. MRI-native feedback

Robust navigation and control require state estimation that remains reliable under MRI's sequence-dependent visibility and phase-dependent imaging constraints. Systematic reviews emphasize that device visibility and tracking performance are sequence-dependent and involve tradeoffs between anatomical clarity and localization robustness.^9,10^ Active steering/tracking approaches illustrate both opportunity and coupling: intravascular catheters controlled using active steering coils,^40^ and active tracking enabling automated slice positioning.^25^ Yet these introduce conductive pathways and tie back to RF heating and RF-safe design constraints.^47,48^

Distributed MR-compatible sensing provides complementary state estimation without electromagnetic interference.^61^ Complementary directions aim to reduce reliance on wired active tracking by developing automated passive tracking pipelines for MR-guided endovascular interventions and integrating them with robotic navigation workflows.^27,28^ Co-designed systems should pursue hybrid state estimation combining MRI-derived localization, distributed sensing, and model-based prediction.

This is also where MRI–robot co-design becomes operational at the imaging level. Real-time MRI progress remains bounded by tradeoffs between temporal resolution, spatial detail, and robustness.^20^ Parallel imaging acceleration is powerful but introduces noise amplification and reconstruction complexity.^64^ When MRI is used as a control feedback channel, these tradeoffs influence not only image quality but control stability and safety.

A robot-aware MRI pipeline should therefore be designed around control-relevant outputs rather than images alone, including stable localization signals, predictable device signatures, reliable latency bounds, and phase-specific imaging modes coordinated with procedural steps.

In practical terms, this suggests workflow-aware sequencing: navigation sequences optimized for update rate and robust localization, followed by targeting sequences optimized for precision and reduced ambiguity near critical anatomy, followed by verification sequences that support deployment confirmation. Reviews of real-time MRI highlight that “real-time” is achieved through design choices that inevitably trade-off with other attributes.^20^ Co-design requires making those tradeoffs explicitly in relation to robotic control needs. This also implies that tracking approaches (active coils, passive markers, image processing) must be chosen not only for accuracy but for how they interact with reconstruction latency, artifact burden, and safety constraints.^25,26,59,60^

### C. The navigation gap

Endovascular robotics is ultimately a navigation problem. The operator (and any assistance layer) must continuously interpret guidewire/catheter progression, anticipate vessel branching, manage friction-dominated tool–vessel interactions, and select safe maneuvers under indirect and often sparse feedback. In MRI-guided settings, this navigation loop is further constrained by variable imaging update rates, sequence-dependent visibility, and workflow-driven phase changes, which together limit the bandwidth and reliability of feedback available for decision-making and motion execution.^9,10,20^

These constraints motivate feedback-efficient control strategies that remain stable and useful when state estimation is intermittent, and uncertainty is high. Rather than relying on high-rate feedback, control architectures must explicitly accommodate phase-dependent imaging regimes and use prediction and constraint enforcement to maintain safety margins during navigation and deployment.^64^

A pragmatic near-term pathway is shared control, where the clinician remains in command while the system stabilizes motion, enforces safety constraints, and reduces sensitivity to transient loss of visibility. Confidence-based shared control in surgical robotics illustrates how supervision and uncertainty can be integrated into assistance strategies in safety-critical settings.^75^ In endovascular manipulation specifically, haptic feedback and dynamic active constraints have been proposed as concrete shared-control mechanisms to stabilize catheterization while preserving operator intent.^13^

In MRI-guided endovascular robotics, however, such assistance strategies must be reformulated for feedback that is intermittent, delayed, and phase-dependent rather than continuously available. This means that the system cannot rely only on what is visible in the latest image, but must also predict what is likely to happen between image updates. In practice, this makes predictive control and state-estimation methods particularly relevant, because they allow the robot to combine previous imaging information, device motion, and procedural context to maintain a working estimate of tool position and behavior until new MRI data arrive.^20,39,64^ Confidence-based shared control and dynamic active constraints would also need to become uncertainty-aware, so that the level of robotic assistance adapts to how reliable and how recent the available MRI feedback is. For example, when localization is uncertain or delayed, the system may need to tighten safety margins or increase operator authority, whereas more reliable updates may allow stronger assistance.^75^ More broadly, MRI-specific control should not treat imaging as a passive measurement source, but as part of the control loop itself. Because MRI acquisition and reconstruction determine what information becomes available and when, future systems could prioritize control-relevant information—such as rapid tip localization, local visibility around a branch point, or phase-specific imaging modes for navigation, targeting, and verification—rather than uniform image quality across the full field of view.^20,25,59,60^

AI-based attention or saliency mechanisms may further strengthen this concept by helping the system identify which image regions or features are most relevant for the current control objective. In MRI-guided navigation, such methods could support phase-dependent imaging regimes by emphasizing different priorities during navigation, branch engagement, or therapy delivery—for example, rapid device localization in one phase and higher-confidence local anatomical context in another. They could also improve prediction between image updates by focusing reconstruction or tracking on the most informative spatial regions, rather than treating all image content as equally relevant for control.

In the longer term, such strategies could be strengthened by digital-twin or simulation-based models of the robot and vasculature, allowing safer prediction between image updates and more robust switching between teleoperation, shared control, and more autonomous behaviors.^39^

Autonomy remains attractive but must be scoped realistically. Reviews emphasize incremental autonomy under meaningful human control, particularly given safety-critical requirements and the limits of perception and state estimation in clinical environments.^76,77^ Learning-based methods for well-defined subtasks (e.g., guidewire feeding) suggest potential for constrained assistance, but they require careful validation and safety-aware system integration—especially when deployed under bandwidth-limited MRI feedback.^78^

A practical way to accelerate progress on navigation assistance while maintaining safety and reproducibility is the use of realistic simulation environments that support algorithm development, benchmarking, and data generation for learning-based assistance and shared control before transfer to physical phantoms and in-scanner validation. A simulation environment developed specifically for robot-assisted endovascular interventions provides a structured foundation for repeatable evaluation of navigation strategies and human–robot interaction under controlled conditions.^39^ Extending such environments to reflect MRI-specific constraints (e.g., intermittent updates and visibility models) would further align simulation benchmarks with MRI-native system requirements.

### D. From constraints to integrated systems

A key next step for the field is to move from describing constraints to building executable MRI–robot co-design workflows that can be reproduced and benchmarked across laboratories. While many enabling components exist—MR-compatible actuation concepts, tracking approaches, and accelerated imaging pipelines—translation will depend on system prototypes that treat RF safety, device visibility, and feedback bandwidth as coupled design targets rather than sequential constraints.^14–18,20,47,48,64,72^

In this context, experimentally grounded robotic testbeds for endovascular intervention provide a practical platform for iterative co-design, allowing objective assessment of how mechanical choices, tracking strategies, and MRI acquisition settings jointly affect clinical task performance.^22,23^ Rather than optimizing isolated metrics (e.g., actuator bandwidth or tracking accuracy), a co-design agenda should prioritize task-level outcomes under realistic imaging and safety envelopes. Representative targets include (i) MRI-native steerability and compliance control that reduce reliance on guidewire exchange while maintaining miniaturization and RF safety margins;^42,54,65,71^ (ii) hybrid state estimation that combines MRI-derived localization with MR-compatible sensing to sustain control when imaging is intermittent or optimized for anatomy rather than device visualization;^27,40,61^ and (iii) feedback-efficient assistance and shared control strategies that stabilize navigation and deployment under variable temporal resolution and uncertainty.^75,76,78^

To avoid “one-off” demonstrations, these directions should be benchmarked with consistent evaluation protocols and clearly reported operating conditions. Practical measures include branch cannulation success, time-to-target, tip positioning error, and repeatability; localization error as a function of imaging update rate; and worst-case heating maps and safety margins under representative sequences and device configurations. This type of benchmarking can also clarify where clinical impact is most likely to emerge first, e.g., in carefully scoped assistance functions, before progressing toward more advanced autonomy.

Finally, clinical risk cannot be abstracted away: guidewire-related complications illustrate why predictable behavior and robust safety constraints matter even under fluoroscopy.^79^ Clinically relevant steerability must be judged by usability and procedural impact, not only feasibility.^80,81^

These relationships are summarized in Table II, which maps key clinical capabilities to the MRI-specific constraints that shape system design and to representative evaluation metrics for benchmarking.

**TABLE II.** Requirements, MRI-specific constraints, and representative evaluation metrics for MRI-guided endovascular robotics. The table links core clinical capabilities relevant to MRI-guided endovascular intervention with the MRI-specific constraints that shape system design and with representative metrics for benchmarking performance. It highlights how instruments, feedback, navigation, and integration must be evaluated not in isolation, but as interdependent domains within MRI-guided endovascular robotics.

Capability (clinical need)	Why MRI changes it	Current limitation (coupled constraint)	Practical evaluation metrics (examples)
Steerability and navigation	MRI limits access/actuation but improves soft-tissue context	Miniaturized actuation constraints; limited distal bandwidth	Branch cannulation success; time-to-target; tip error; repeatability
Stiffness/compliance modulation	Helps safety/precision when imaging is intermittent	Packaging limits; may alter visibility/artifact profile	Stiffness range; response time; steering retention; contact force bounds
Device visibility and tracking	Visibility is sequence-dependent; artifacts can obscure anatomy	Active tracking adds RF/heating + integration burden	Localization error; update latency; obscuration score; failure rate under motion
RF safety	RF coupling can heat long devices	Heating depends on geometry/sequence; must be designed-in	ΔT vs sequence; worst-case heating map; regulatory compliance
Feedback-efficient control	Imaging tradeoffs affect control stability	Phase-dependent updates; variable visibility; uncertain state	Stability margins; safe error envelopes; success at reduced update rate
Workflow feasibility	Bore constraints + team choreography shape adoption	Setup/access constraints; sequence transitions disrupt usability	Total time; setup time; mode switches; operator workload scores

### E. Translational milestones

The four research axes outlined above are likely to mature on different but interdependent timescales, and their translational path is best understood as a staged progression rather than a single technological leap. In the near term (3–5 years), the most realistic milestones are expected at the level of enabling components and controlled preclinical validation. For MRI-native instruments, this includes improved MR-safe actuation concepts, RF-safe materials and architectures, and steerable devices with more predictable mechanical behavior at clinically relevant scales. For MRI-native feedback, near-term progress is likely to come from more robust tracking and visualization strategies, including passive and active tracking pipelines, robot-aware MRI acquisition, and hybrid state-estimation approaches that combine imaging with complementary sensing. For navigation, the most achievable goals are feedback-efficient shared-control strategies, model-based state estimation under intermittent imaging, and simulation environments that support repeatable development and benchmarking. At the systems level, near-term milestones include standardized phantom and *ex vivo* validation protocols, clearer safety reporting, and benchmarking procedures that allow more meaningful comparison across devices and workflows.

In the mid-term (5–10 years), progress should shift from isolated components toward partially integrated MRI-native systems. In this phase, MRI-native instruments will need to demonstrate improved miniaturization together with clinically meaningful steerability, stiffness control, and RF-safe performance under representative imaging conditions. MRI-native feedback should mature into workflow-aware pipelines capable of supporting control-relevant outputs with predictable latency and sufficient robustness across procedural phases, while navigation strategies should evolve from shared assistance toward more adaptive, task-level support under delayed and phase-dependent measurements. A key milestone in this interval will be demonstrating that MRI-guided robotic workflows can provide practical procedural value beyond proof-of-concept feasibility.

In the long term (>10 years), the field should move toward clinically usable MRI-native endovascular robotic platforms in which instruments, feedback, navigation, and workflow are co-designed as a unified system rather than sequentially combined. Such platforms would need to support reliable MRI-safe manipulation, robust state estimation, workflow-compatible operation, and carefully scoped autonomy or shared autonomy where clinically justified. These stages are linked by several critical dependencies: navigation depends on robust MRI-native feedback and state estimation, integration depends on reproducible validation and benchmarking infrastructures, and clinically meaningful autonomy depends on reliable instruments, control, and workflow-compatible system architectures. These dependencies suggest that translation will be driven not by a single breakthrough, but by coordinated maturation across the four axes.

## VI. OUTLOOK: FROM FEASIBILITY TO MRI-NATIVE SYSTEMS

MRI-guided endovascular robotics remains an emerging but still fragmented field, and significant technological and methodological challenges must be addressed before these systems can be widely adopted in clinical practice. While representative platforms, MRI-compatible devices, and MRI-native feedback frameworks are beginning to emerge, many current solutions still represent adaptations of technologies originally developed for fluoroscopy-guided interventions. As discussed throughout this paper, these approaches frequently encounter fundamental limitations related to miniaturization, controllability, and integration within the MRI environment.

Future research will likely benefit from a shift toward MRI-native design principles, where robotic systems are conceived from the outset to operate within the unique electromagnetic and imaging environment of MRI. Rather than merely mitigating scanner constraints, such approaches aim to integrate MRI-derived sensing and imaging feedback directly into robotic design and control. This paradigm may enable new classes of robotic devices that achieve both improved compatibility with MRI systems and enhanced functionality for endovascular navigation.

The most promising directions are those that advance the four coupled axes outlined in this paper: MRI-native instruments, MRI-native feedback, navigation under bandwidth-limited imaging, and integrated systems built through workflow-aware validation and benchmarking. Progress will depend not only on device performance, but also on the ability of these systems to operate within realistic interventional workflows and demonstrate clear advantages over established fluoroscopy-based approaches.

Ultimately, the convergence of advances in MRI technology, soft robotics, smart materials, and intelligent control creates an opportunity to rethink endovascular robotics around the capabilities and constraints of the MRI environment itself. Rather than adapting fluoroscopy-era instruments to operate inside the scanner, the next generation of systems will likely emerge from MRI–robot co-design, where actuation, sensing, imaging, and control are developed as a unified architecture. The research axes outlined in this Perspective provide a practical roadmap for this transition toward MRI-native endovascular robotic systems. By embracing this MRI-native perspective, future robotic platforms may enable safer, radiation-free, and more precise image-guided interventions, opening new possibilities for minimally invasive therapies.

## Data Availability

The data that support the findings of this study are available from the corresponding author upon reasonable request.
